# Proteoglycan 4 (PRG4)/Lubricin and the Extracellular Matrix in Gout

**DOI:** 10.3390/gucdd1030012

**Published:** 2023-07-11

**Authors:** Khaled A. Elsaid, Gregory D. Jay, Ru Liu-Bryan, Robert Terkeltaub

**Affiliations:** 1 Department of Biomedical and Pharmaceutical Sciences, Chapman University, Irvine, CA 92618, USA; 2 Department of Emergency Medicine, Alpert Medical School of Brown University and School of Engineering, Brown University, Providence, RI 02903, USA;; 3 VA San Diego Healthcare System, San Diego, CA 92161, USA;

**Keywords:** PRG4, lubricin, gout, collagen type II, synovium

## Abstract

Proteoglycan 4 (PRG4) is a mucinous glycoprotein secreted by synovial fibroblasts and superficial zone chondrocytes, released into synovial fluid, and adsorbed on cartilage and synovial surfaces. PRG4′s roles include cartilage boundary lubrication, synovial homeostasis, immunomodulation, and suppression of inflammation. Gouty arthritis is mediated by monosodium urate (MSU) crystal phagocytosis by synovial macrophages, with NLRP3 inflammasome activation and IL-1β release. The phagocytic receptor CD44 mediates MSU crystal uptake by macrophages. By binding CD44, PRG4 limits MSU crystal uptake and downstream inflammation. PRG4/CD44 signaling is transduced by protein phosphatase 2A, which inhibits NF-κB, decreases xanthine oxidoreductase (XOR), urate production, and ROS-mediated IL-1β secretion. PRG4 also suppresses MSU crystal deposition in vitro. In contrast to PRG4, collagen type II (CII) alters MSU crystal morphology and promotes the macrophage uptake of MSU crystals. PRG4 deficiency, mediated by imbalance in PRG4-degrading phagocyte proteases and their inhibitors, was recently implicated in erosive gout, independent of hyperuricemia. Thus, dysregulated extracellular matrix homeostasis, including deficient PRG4 and increased CII release, may promote incident gout and progression to erosive tophaceous joint disease. PRG4 supplementation may offer a new therapeutic option for gout.

## 1. Introduction

The pathophysiology of gout, the most common cause of inflammatory arthritis, includes intersections between genetics, urate homeostasis, innate immunity, and diseases of metabolism, and renal and cardiovascular function [1–3]. Urate is particularly limited in its solubility in joint tissues. Additionally, sustained hyperuricemia is a primary risk factor for deposition of monosodium urate (MSU) crystals in synovial joints, and a variety of soft tissues including bursae and tendons [2–4]. Inflammatory joint disease in gout is chronic, and characteristically punctuated clinically by bouts of acute and excruciatingly painful arthritis and soft tissue inflammation.

Acute inflammation in gout is driven via recognition of urate crystals by tissues’ monocytes/macrophages and their subsequent phagocytosis [5,6]. Activation of the NOD-, LRR- and pyrin domain-containing protein 3 (NLRP3) inflammasomes in tissue phagocytes underlies their acute response to urate crystals, resulting in the recruitment of pro-caspase-1 and its conversion to active caspase-1 [6]. Active caspase-1 converts pro-interleukin-1 beta (pro-IL-1β) to mature IL-1β, which drives inflammation in gout [4]. Synovium is comprised of a surface layer, the intima and an underlying subintima [7,8]. The intima of normal synovium is one to three cell layers thick, with two cell types: fibroblast-like synoviocytes and macrophages [7,8]. Synovial macrophages comprise heterogenous populations whose functions include the clearance of cell debris and foreign bodies, tissue immune surveillance, and the resolution of inflammation [9,10]. Importantly, cartilage-derived damage-associated molecular patterns (DAMPs) activate macrophages, and this contributes to the pathophysiology of synovitis [11]. In this review, we comprehensively summarize the current understanding of how synovial joint extracellular matrix homeostasis and specific synovial fluid (SF) components impact gout pathophysiology. Our primary focus is on how proteoglycan 4 (PRG4) limits gouty arthritis, with attention also given to the contrasting effects of cartilage-derived collagen type II (CII).

## 2. Proteoglycan 4 (PRG4)/Lubricin: Articular Localization, Structure, Regulation and Biological Activity

The *PRG4* gene is alternatively spliced and is responsible for the mucinous glycoproteins lubricin and superficial zone protein (SZP). Lubricin is secreted by synovial type B fibroblasts, and SZP by superficial zone chondrocytes [12–15]. PRG4 is a major component of synovial fluid (SF) and is localized on the surface of articular cartilage, where it functions as a boundary lubricant at near-zero sliding speeds and prevents cell and protein adhesions [13,16–18]. The boundary-lubricating property of PRG4 prevents friction-induced mitochondrial dysregulation and chondrocyte apoptosis [19,20]. PRG4 is also found in the synovium [21]. The full length synovial form of PRG4/lubricin has a semi-rigid structure. The protein core has 1404 amino acids with N and C termini and a central mucin domain that is heavily glycosylated via O-linked β(1–3) Gal-GalNac oligosaccharides (which account for ~50% of the mucin weight) and is responsible for its boundary-lubricating function [16,22] (Figure 1). The globular N- and C-termini of PRG4 may be involved in multiple biological functions [16]. In the N-terminus, there is a heparin-binding site, a chondroitin sulfate chain, and a somatomedin B-like domain [15,16]. The C-terminus contains a hemopexin-like domain [15,16]. In both domains, PRG4 has greater than 40% sequence similarity with vitronectin, though PRG4 has a unique repeating motif of KEPAPTT in which *O*-glycosylations are found [16]. Either the N- or C-terminus or both mediate PRG4’s anchoring to surfaces, which results in a brush-like conformation that provides optimal boundary lubrication [23]. The N-terminus is also the site of disulfide bonding, wherein PRG4 exists as monomers, dimers, and multimers, with improved boundary lubrication observed with multimeric PRG4 [24,25]. The concentration of PRG4 is normally high in SF (200 to 400 μg/mL) [26].

The loss of function mutation in *PRG4* is evident in the autosomal recessive disease camptodactylyl-arthropathy-coxa vara-pericarditis (CACP), a rare juvenile onset arthropathy [21,27]. The murine *Prg4* knockout model displays key features of CACP disease, and the joints of *Prg4* null animals exhibit synovial inflammation, hyperplasia, and fibrosis in addition to cartilage surface damage and chondrocyte apoptosis, which may not be completely reversed by *Prg4* re-expression [21,28–30]. Human studies that have examined PRG4 SF levels in different cohorts of joint injuries, moderate OA, and advanced OA have reported either a decrease, no change, or an increase in SF PRG4 levels in reference to healthy subjects [31]. Studies that reported a decrease in SF PRG4 levels were more likely to include patients with anterior cruciate ligament or meniscal tears within one year of injury, whereas four out of five studies that reported an increase in SF PRG4 levels included patients with advanced OA [31]. It is unclear whether the different assays used in these studies to quantify SF PRG4 could differentiate between full-length and degraded protein.

Multiple studies in animals showed that cartilage, synovial *PRG4* expression, or a combination thereof were reduced in mouse, rat and guinea pig models of naturally occurring and posttraumatic OA (PTOA) [32–36]. Using in vitro models, *PRG4* expression in chondrocytes and synoviocytes was shown to be reduced by IL-1β and tumor necrosis factor (TNF), and increased by transforming growth factor beta 1 (TGF-β1) [37–41]. Furthermore, PRG4 is proteolytically degraded by multiple enzymes, e.g., elastase, and cathepsins B and G [36,42]. A summary of studies that showcase the disease-modifying effects of native and recombinant PRG4 in pre-clinical PTOA models is presented in Table 1 [43–50]. In addition, *Prg4* gene therapy is efficacious in mitigating murine age and injury-related OA development [51–53].

Biologically, PRG4 binds transmembrane CD44 receptors and competes with high-molecular-weight hyaluronic acid (Hyaluronan) to do so. As a consequence of preferentially binding CD44, PRG4 reduces the mitogen-activated proliferation of mouse *Prg4*^−*/*−^ synoviocytes and human synoviocytes from patients with OA and rheumatoid arthritis (RA) [54,55]. In OA synoviocytes, recombinant human PRG4 (rhPRG4) treatment reduces NF-κB nuclear translocation via inhibition of IκBα phosphorylation [55], with downstream reduction of the expression of matrix metalloproteinases (MMP1, MMP3, MMP9, MMP13) and cytokines IL-6 and IL-8 [55]. PRG4 also binds to the toll-like receptor (TLR) family of pattern recognition receptors [56,57], and PRG4 suppresses activation of TLR2 and TLR4 receptors by DAMPs in SF aspirates from patients with OA [57].

PRG4 plays a significant role in regulating synovial macrophages. In *Prg4*-deficient mice, macrophages accumulate in synovial tissues with age [58], while the tissue resident fraction is reduced. Total macrophages in the synovium skew to a predominantly CD86+ pro-inflammatory phenotype, and away from the CD206+ anti-inflammatory phenotype [58]. *Prg4* re-expression in mice reduces total macrophages in synovial tissues and re-establishes homeostasis with an enrichment in anti-inflammatory CD206+ synovial macrophages [58]. Furthermore, *Prg4* deficiency appears to prime acute synovitis, as demonstrated by enhanced inflammatory macrophage recruitment [58]. Interestingly, synovial macrophage depletion in otherwise *Prg4*-deficient mice reduces synovial hyperplasia and synovial fibrosis [58]. These collective observations support PRG4’s immunomodulatory and anti-inflammatory roles in the joint.

## 3. The PRG4/Lubricin Receptor CD44 and Protein Phosphatase 2A Signal Transduction

CD44 is a single-pass heavily glycosylated transmembrane receptor that is alternatively spliced to generate various isoforms [59]. CD44 is widely expressed, including by cells of the immune system, and CD44 contributes to signaling by a broad array of microenvironmental constituents, e.g., not only CD44 ligands, but also cytokines and growth factors [60]. Beyond ligand binding by the CD44 extracellular domain (ECD), the transmembrane domain of CD44 interacts with co-factors and adaptor proteins, and directs lymphocyte homing [59,60], whereas the intracellular domain binds different signaling partners and may translocate to the nucleus to activate gene transcription involved in cancer cell survival and metastasis [61–63]. CD44 ligands include hyaluronan, osteopontin, collagens, and MMPs, and the role of CD44 in mediating cell migration and growth is ligand-dependent [64–66]. CD44 has additional roles in regulating inflammation and immune responses via its role as a phagocytic receptor [67]. CD44 functions to clear apoptotic cells and pathogens [68,69]. CD44 is sufficient by itself to mediate the phagocytosis of large particles, and it also contributes to phagocytosis as a coreceptor that tethers foreign particles which are internalized using other molecules, e.g., phosphatidylserine [67].

CD44 has been associated with multiple central signaling pathways including Rho GTPases, Ras-MAPK, PI3K/AKT, and phosphatase pathways [70]. The signaling pathway that is predominantly activated is CD44 isoform-, cell- and ligand-specific [70]. CD44 engagement by hyaluronan triggers activation of downstream effectors, e.g., AKT, ERK, and protein phosphatase 2A (PP2A) [71]. PP2A is a highly conserved member of the serine/threonine phosphatase family of enzymes that regulate the signal transduction pathways of most cellular processes [72]. The holozyme is a heterotrimer of catalytic, regulatory, and structural subunits, which when assembled generate multiple structurally and functionally distinct enzyme complexes [73]. The composition of the holozyme determines substrate specificity and cellular localization, while post-translational modification of PP2A’s catalytic subunit by methylation or phosphorylation regulates its enzymatic activity [74]. Dysregulated cellular PP2A activity has been associated with multiple diseases including neurodegenerative disorders, diabetes, cancer, heart disease, and inflammation [75–78]. In inflammation, PP2A restrains the phosphorylation of the NF-κB signaling pathway via dephosphorylation of IKKβ, IκBα, and RelA [79]. PP2A has a role in regulating the innate immune response of macrophages; enzyme inactivation via targeted deletion of the catalytic subunit increases TNF expression in LPS-stimulated murine bone marrow-derived macrophages (BMDMs) [80].

## 4. CD44 Receptors and MSU Crystals: The Role of PRG4 as a Ligand and PP2A as a Signal Transducer

We identified CD44 as a major, biologically significant, phagocytic receptor for MSU crystals [81]. We also discovered that its ligand, PRG4, inhibits MSU crystal phagocytosis by human and murine macrophages through a CD44-dependent mechanism [82], suppresses xanthine oxidoreductase (XOR) expression and urate production by murine macrophages [83], and ameliorates inflammation in two pre-clinical rodent models of acute gout [82,84]. PP2A inactivation in human monocytes and murine macrophages as a result of MSU crystal exposure was determined to be permissive for NLRP3 inflammasome activation and IL-1β release [85].

Key findings that support the roles of CD44 as a phagocytic receptor, PRG4 as an anti-inflammatory therapeutic in acute gout, and PP2A’s involvement in gout pathophysiology are presented in Table 2.

PP2A dysfunction, under different conditions that include inflammation, might be related to the accumulation of reactive oxygen species (ROS). The ROS, in turn, inactivate the enzyme via mechanisms that include tyrosine phosphorylation, leucine carboxy methylation, and the dissociation of the enzyme’s catalytic subunit [87–89]. We identified XOR in macrophages as a major source of ROS production in MSU crystal-stimulated BMDMs [90]. This is in line with a previous study, in which ROS generated from XOR were identified as a significant driver of NLRP3 inflammasome activation and IL-1β production [91]. XOR-derived ROS accumulation inactivates PP2A in macrophages [90]. In addition, a PP2A-activating drug treatment inhibits XOR expression, ROS and urate production and stimulates the expression of nuclear factor erythroid 2-related factor 2 (Nrf2), a key transcriptional activator of antioxidant response in macrophages [90,92]. Upon pharmacological activation of PP2A in vivo, the recruitment of neutrophils and inflammatory classical monocytes is reduced in a peritoneal model of acute gout, and this effect is associated with enhancement in anti-inflammatory nonclassical monocytes’ ingress into the site of inflammation [90].

The role of PP2A in gout inflammation at the macrophage cellular level is schematized in Figure 2. According to our model, the PRG4/CD44/PP2A circuit functions to suppress XOR expression and hence the production of urate and ROS. Addition to the extracellular pool of urate can promote MSU crystal deposition, and XOR activity-derived ROS promote activation of the NLRP3 inflammasome. Dysfunction of the circuit can occur at the level of the ligand, PRG4, which is downregulated by IL-1β and/or proteolytically degraded. In addition, dysfunction can occur at the level of the intracellular signal transducer, wherein PP2A is inactivated by XOR-derived ROS. Hence, enhancing intracellular PP2A activity may represent a new target area in gout management.

### 4.1. The PRG4/CD44/PP2A/XOR Circuit and Regulation of Synovitis

Cumulative evidence, derived from both in vitro and in vivo experiments, points to a native PRG4/CD44/PP2A/XOR regulatory circuit that is fundamental to synovial tissue homeostasis. The failure of this circuit is permissive for acute gout inflammation, which if ineffectively resolved, may potentially lead to chronic synovitis and joint destruction [93,94]. The conceptual framework of this regulatory model is presented in Figure 3. Circuit failure can occur at the ligand level (PRG4) via a downregulation at the gene level and/or proteolytic degradation by enzymes, e.g., cathepsin G [83]. Failure at the receptor level occurs via upregulation of CD44 expression on macrophages by inflammatory cytokines shifting the equilibrium towards unoccupied CD44 receptors. Failure of the signaling transducer occurs via inactivation of PP2A by ROS, and the downstream effect of the regulatory circuit is an enhancement of XOR expression driving M1 polarization and secretion of cytokines and chemokines, resulting in the promotion of acute gout inflammation. Conversely, anti-inflammatory cytokines, e.g., TGF-β, which is produced by inflammatory and anti-inflammatory macrophages [95] to promote the resolution of inflammation, upregulates PRG4 expression by synoviocytes [30]. The increase in PRG4 levels in the synovial microenvironment “cloaks” the CD44 receptor and arrests active MSU crystal phagocytosis. Furthermore, PRG4 binding to CD44 results in the activation of PP2A, which inhibits the NF-κB signaling pathway, M1 macrophage polarization, and shifts the balance of resident macrophages towards the anti-inflammatory M2 phenotype characterized by secretion of anti-inflammatory cytokines, e.g., IL-10. As a result, nonclassical monocytes entry into synovium surges, and such monocytes preferentially differentiate to M2 macrophages, which facilitate tissue repair and the resolution of inflammation.

### 4.2. PRG4 Deficiency Associated with Erosive Gouty Arthritis Independent of Hyperuricemia

Characterization of unusual and severe cases of disease is highly informative for pathophysiology. In our work, we characterized a central pathogenic role of PRG4 deficiency in a healthy 22-year-old white female diagnosed with an aggressive, destructive autoinflammatory disease meeting the criteria for gout, despite a normal serum urate level (5 mg/dL) [83]. Imaging and SF examination confirmed the presence of MSU crystals and upon treatment with allopurinol (400 mg/day), her serum urate level decreased to ~2 mg/dl, and this was associated with a marked reduction in gout flares. Whole genome sequencing, whole blood RNA sequencing and quantitative proteomics revealed the presence of an NLRP3 V198M variant, a low serum PRG4 level, and genomic and serum proteomic interactome network changes that favored increased PRG4 degradation. The changes included a predicted deleterious mutation in the protease inhibitor ITIH3, and low serum levels of the protease inhibitors thrombospondin1 and Serpin B6. ELISA and proteomics studies identified that the PRG4 level in the patient’s serum needed to be attenuated, and very low serum PRG4 was also seen in 5 out of 18 patients with gout. Whole-blood PRG4 mRNA was not decreased in the patient compared to her parents or healthy controls. Follow-up studies demonstrated that the activity of the neutrophil-released protease cathepsin G activity was increased in patient’s serum, and in multiple gout patients, while undetected in healthy controls. Furthermore, the activated neutrophil-released cathepsin G co-activator lactoferrin was increased in the patient’s serum, and also elevated in a cohort of gout patients relative to matched non-gout controls. Strikingly, the serum level of the cathepsin G substrate amyloid precursor protein (APP) was attenuated in the patient and her parents. While this presentation is rare, it provides strong corroborating evidence for the central implication of PRG4 in gout disease pathophysiology. Moreover, the study provided evidence for multiple potential new biomarkers for susceptibility to both incident and progressive, erosive gout, such as an “interactome” network of physically interacting serum proteins cited above that mediate proteolysis of PRG4, as well as phagocyte inflammatory responses and NETosis, which itself is implicated in tophus generation and the resolution of acute gouty inflammation [96].

## 5. Cartilage Proteins and Gout Pathogenesis

DAMPs comprise a heterogenous group of cartilage fragments, extracellular matrix (ECM) proteins, secreted intracellular proteins, and crystals including MSU crystals, which play a significant role in mediating synovial inflammation [97–99]. DAMPs bind to pattern recognition receptors including membrane-bound receptors, e.g., the TLRs or receptors for advanced glycation end products (RAGE), and cytoplasmic receptors such as NOD-like receptors (NLRs) [97,98]. DAMPs are broadly classified based on their origins as either intracellular or extracellular. Intracellular DAMPs are immunogenic molecules, released from apoptotic cells such as S100A9, a member of the calcium-binding S100 proteins that stabilize the cytoskeleton [98]; high-mobility group box protein 1 (HMGB1), a nuclear protein that facilitates transcription factor and nucleosome stability; and urate [97,98]. Extracellular DAMPs are ECM proteins and their breakdown fragments. Native ECMs that act as DAMPs include CII, biglycan and fibronectin [100–102]. Common among these native ECMs is their ability to activate the p38 and NF-κB signaling pathways via binding to the discoidin domain receptor (DDR2) (CII) [100] and TLR2/TLR4 (biglycan and fibronectin) [101,102]. ECM fragments that act as DAMPs include aggrecan 32-mer fragment, fibronectin fragments, low-molecular-weight hyaluronan, the Coll2-1 CII peptide, and the N-terminal telopeptide of CII [97,103–106]. Similarly, TLR2 and TLR4 contribute significantly to the pro-inflammatory signaling of these breakdown DAMPs [103–106].

Few studies have investigated the contribution of cartilage-derived DAMPs to MSU crystal inflammation in the joint [107,108]. This is surprising, given the significant association between OA and gout, where OA joints have a greater clinical chance of developing gout, suggesting overlapping pathophysiological mechanisms [109]. Chhana et al. studied the impact of normal and diseased cartilage homogenates on urate crystal formation in vitro, and found that both normal and diseased cartilage increase the total mass of MSU crystals and result in the formation of shorter crystals [107]. To identify the cartilage factor(s) responsible for formation of shorter urate crystals, the authors repeated the MSU crystallization assays in the presence of different cartilage factors, and only CII was found to cause the formation of shorter MSU crystals. MSU crystals grown with cartilage homogenates produce significantly higher IL-8 compared to control MSU crystals, suggesting a higher potential to induce inflammation. In another study, and using Raman spectroscopy, Xu et al. discovered that in gout patients with cartilage injuries, CII is enriched on MSU crystals [108]. Full-length CII also causes the formation of shorter crystals and influences the alignment of crystal bows, resulting in greater phagocytosis by macrophages with downstream ROS generation, chemokine and pro-inflammatory cytokine expression [108]. Interestingly, PRG4 prevents MSU crystal formation in vitro [83], and being a major component in SF, PRG4 potentially has a biophysical role against formation of particulate danger signals in the SF. Since CII-enriched MSU crystals are more readily phagocytosed by macrophages, and since the CII-MSU complex enhances TLR2 and TLR4 expression by macrophages, PRG4 may act to counteract the effect of CII via inhibition of MSU crystal formation, phagocytosis, and the TLR2/TLR4-NF-κB signaling pathway. The posited opposing roles of CII and PRG4 in the context of MSU crystal formation, phagocytosis, and synovial macrophage activation are presented in Figure 4.

## 6. Future Areas for Research

The discovery of a PRG4/CD44/PP2A/XOR circuit that acts in the joint to maintain synovial tissue homeostasis raises interesting new questions:

- What is the importance of PRG4 to synovial macrophage barrier integrity in the joint [110] in the context of acute urate crystal inflammation?
- Does disruption to the integrity of this barrier layer increase the likelihood of a gout flare and/or increase its severity?
- To what extent does XOR regulate synovial macrophage plasticity in the synovium, and in which patients will the urate produced in the joint increase the disease burden? This is quite relevant, since an erosive form of gout with urate crystal deposition was observed in an otherwise normouricemic patient [83].
- Does SZP regulate urate crystal deposition on cartilage surfaces and affect XOR expression via superficial zone articular chondrocytes?
- What is the impact of changes in the *O*-glycomap of PRG4 or its degradation fragments on urate crystal formation and synovial macrophage priming in gout? Potentially, there is a pro-inflammatory effect that is a consequence of these changes, since in late-stage OA, synovial PRG4 may have increased unsialylated core 1 *O*-glycans, which compromise its ability to bind galectin-3, a pro-inflammatory mediator [111]; meanwhile, truncated *O*-glycans of PRG4, commonly found in OA, promote proinflammatory cytokine production and may exacerbate synovitis [112]. Moreover, tryptase-mediated cleavage of PRG4 in OA SF activates TLR2 and TLR4 receptors [113].

## 7. Summary and Conclusions

The mucinous glycoprotein, PRG4, has a multifaceted role in the joint, including boundary lubrication and synovial homeostasis. PRG4 binds different receptors, e.g., CD44, TLR2, and TLR4, and suppresses pro-inflammatory and pro-fibrotic signaling pathways. PRG4 suppresses urate crystal uptake by mouse macrophages, human monocytes, and macrophages, and prevents NLRP3 inflammasome activation and the secretion of mature IL-1β. Mechanistically, this is accomplished by binding and cloaking the CD44 receptor, a direct mediator of urate crystal phagocytosis by macrophages. PRG4-CD44 interaction also activates intracellular PP2A signaling that inhibits NF-κB activation and suppresses the levels of XOR, urate and ROS in inflammatory macrophages. PRG4 deficiency was implicated in an erosive, destructive form of autoinflammatory arthritis that met the criteria for gout in an otherwise healthy, normouricemic young female adult. rhPRG4 ameliorates acute gout inflammation in mouse and rat models, and reduces IL-1β secretion from gout PBMCs, which is comparable to the effect of IL-1Ra. PRG4 biology and turnover may provide novel biomarkers for the risk of incident and progressive gout. Furthermore, changes in PRG4 expression and turnover may help explain the linkages of gout to NETosis and to osteoarthritis. Since PRG4 acts upstream of the disease onset and progression processes in gout, PRG4 has the potential to provide a new approach for the management of acute and chronic gouty arthritis, including using engineering means to protect and supplement PRG4 for refractory erosive and tophaceous gout.

## Figures and Tables

**Figure 1.**
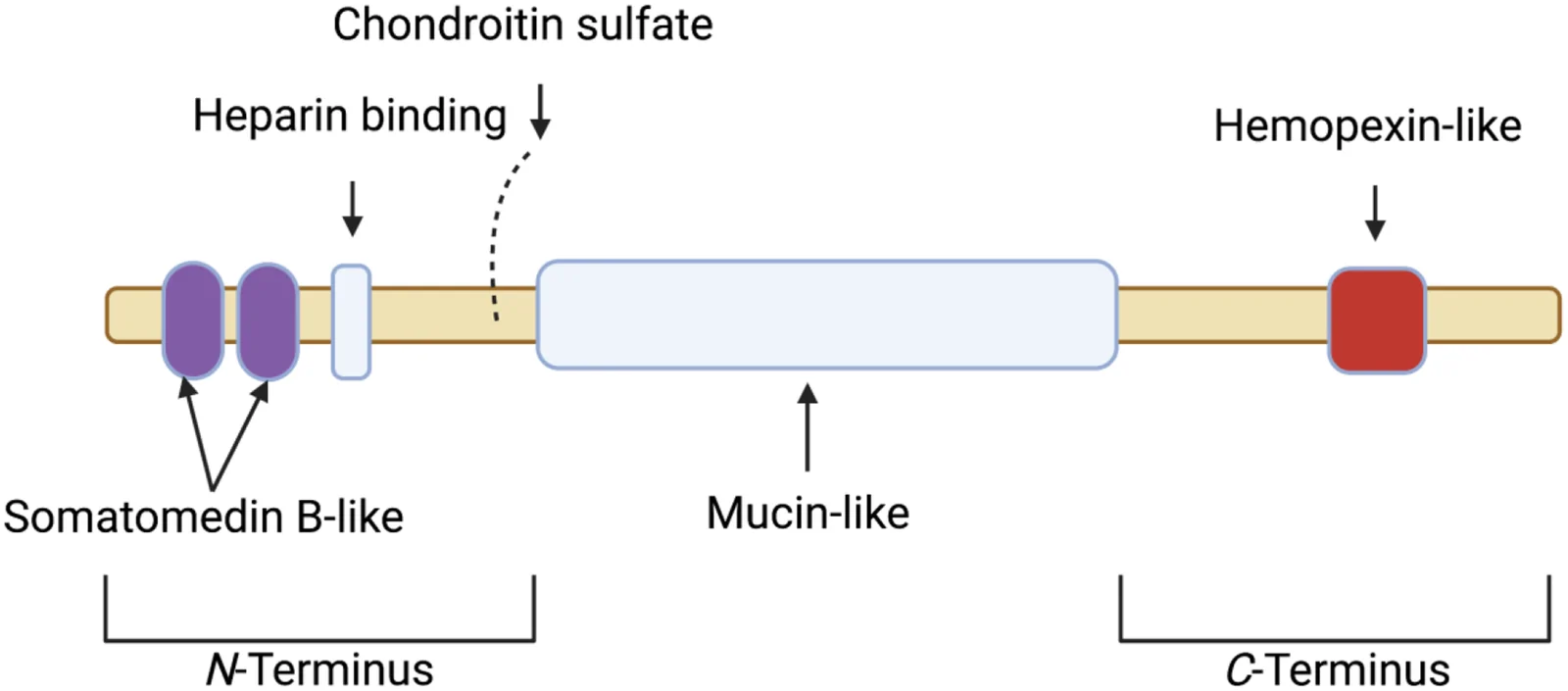
Schematic depicting the various motifs within the full length synovial 1404 amino acid proteoglycan 4 (PRG4) polypeptide. The N-terminus contains a somatomedin B-like domain, a heparin binding site and a chondroitin sulfate chain. The N-terminus is also the site for the disulfide bonding of PRG4 monomers. The central domain is mucin-like, and is responsible for boundary lubrication. The C-terminus contains a hemopexin-like domain.

**Figure 2.**
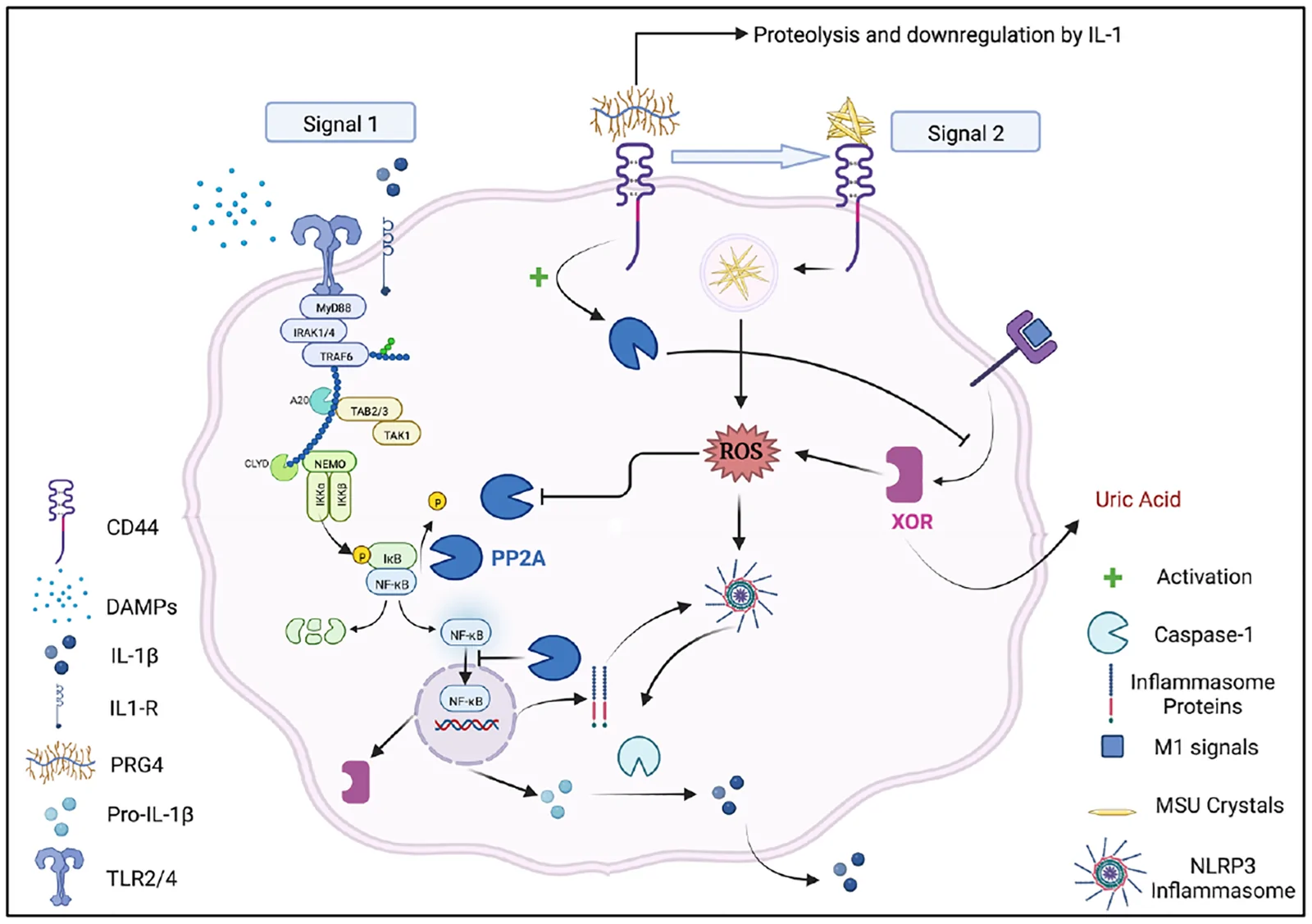
Macrophage activation by urate crystals and the role of the PRG4/CD44/PP2A axis. Mechanistic overview of the regulation of XOR-derived reactive oxygen species (ROS) generation in macrophages by protein phosphatase 2A (PP2A), and corresponding PP2A’s inactivation by XOR-derived ROS as the basis for PP2A’s role in gout inflammation. Resident macrophages are primed by IL-1β or by DAMPs (Signal 1). Both stimuli independently activate the NF-κB pathway, resulting in its nuclear translocation and expression of pro-IL-1β, inflammasome proteins, and XOR. PP2A regulates the NF-κB pathway via dephosphorylation of IκBα, which prevents the translocation of NF-κB. Under inflammatory conditions, PRG4 is downregulated at the gene level and/or proteolytically degraded, thus compromising the integrity of the PRG4/CD44/PP2A signaling pathway. Unoccupied CD44 receptors bind MSU crystals, which are then internalized by the macrophages (Signal 2). MSU crystals and macrophage M1 (pro-inflammatory) signals, including IL-1β and DAMPs, upregulate XOR expression, which results in increased XOR activity and production of urate; XOR activity-derived ROS can activate the NLRP3 inflammasome and inactivate PP2A. Inflammasome assembly activates caspase-1, which in turn converts pro-IL-1β to mature IL-1β.

**Figure 3.**
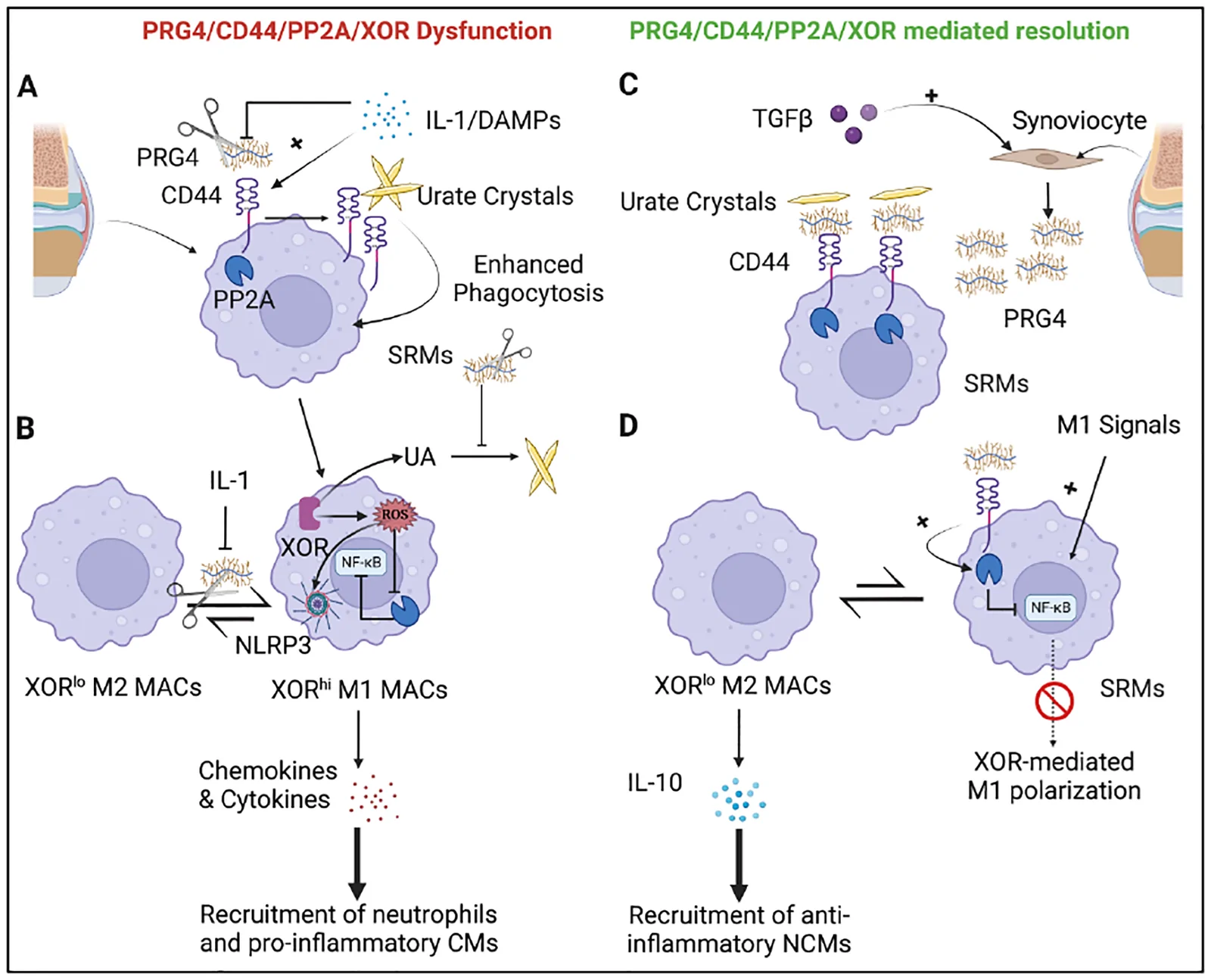
Foundation of synovial tissue homeostasis by the PRG4/CD44/PP2A/XOR circuit. The failure of this circuit includes (**A**) Inhibition of PRG4 synthesis and/or its degradation by proteases (denoted by scissors) in the setting of acute inflammation, while simultaneously increasing CD44 expression on synovium-resident macrophages (SRMs). Unoccupied CD44 mediates urate crystal uptake and downstream XOR expression. IL-1β and GM-CSF (gout mediators) also induce XOR in macrophages (MACs). (**B**) XOR drives SRMs towards XOR^hi^ M1 MACs, while PRG4 deficiency exacerbates M1 MAC accumulation. XOR products: urate (UA) is secreted into the joint space and promotes crystal deposition/growth, and reactive oxygen species (ROS) activate the NLRP3 inflammasome and inhibit PP2A activity. PRG4 also interferes with MSU crystal formation [83]. M1 MACs release chemokines and pro-inflammatory cytokines that recruit neutrophils and classical monocytes (CMs). Circuit reconstitution effectively resolves acute inflammation using the following mechanisms: (**C**) TGF-β upregulates PRG4, resulting in “cloaking” of CD44 on SRMs and inhibition of urate crystal phagocytosis. (**D**) PRG4 binding CD44 activates intracellular PP2A, inhibits NF-κB, and prevents M1 polarization. XOR^lo^ M2 MACs secrete IL-10, which promotes the viability of and preferential differentiation into anti-inflammatory/wound-healing M2 MACs of the non-classical monocytes (NCMs) that have surged into the acutely inflamed gouty joint.

**Figure 4.**
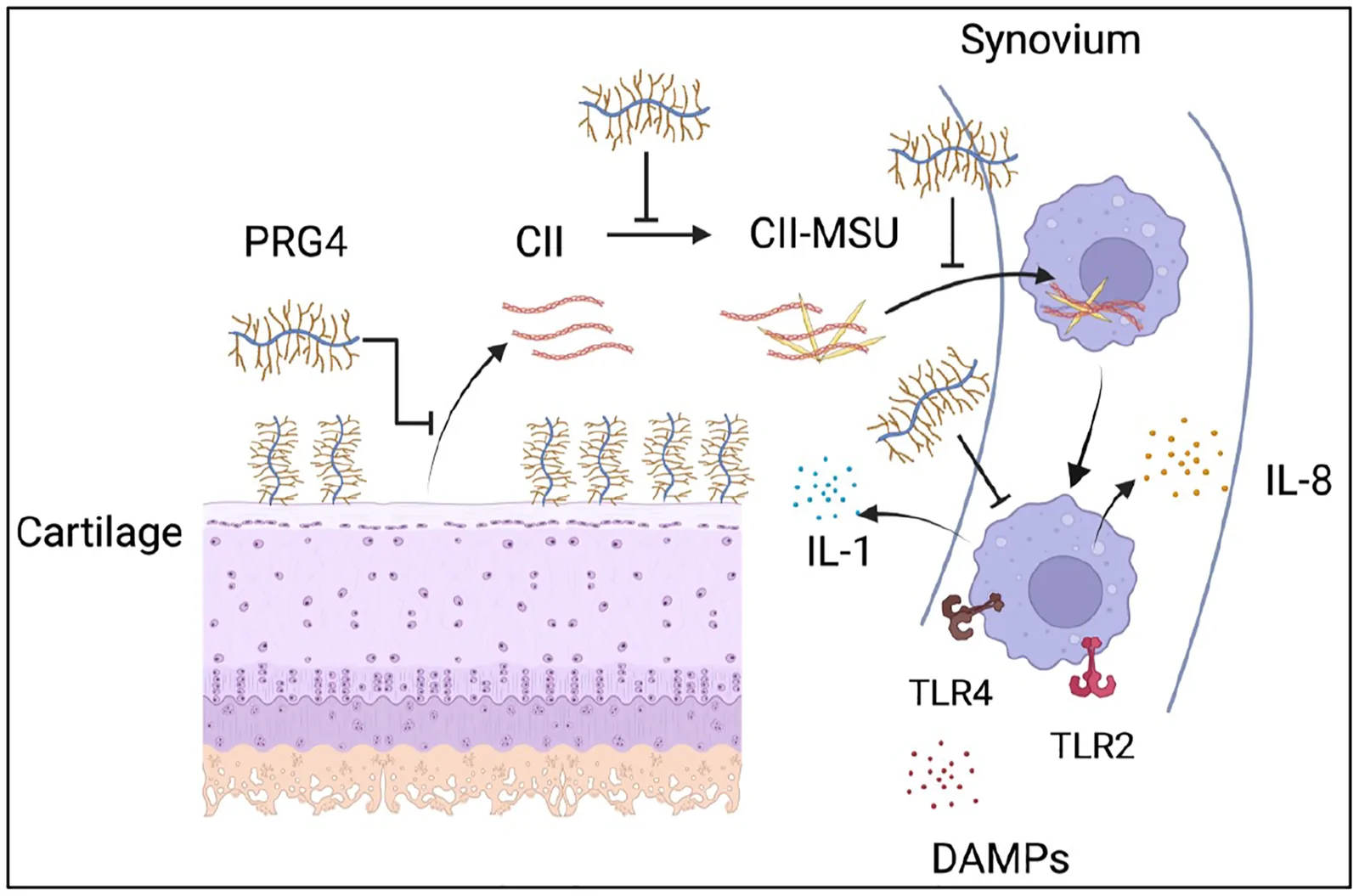
A hypothetical model of proteoglycan 4 (PRG4) and collagen type II (CII) in gout pathogenesis. We posit that PRG4 and CII have opposing roles in the joint environment. CII fragments are released from cartilage in response to mechanical injuries and MMP-mediated proteolysis. PRG4 is known to prevent cartilage surface damage and degeneration via biophysical and biological mechanisms. CII fragments initiate the formation of shorter MSU crystals, while PRG4 inhibits MSU crystallization. The CII-MSU crystals are more readily phagocytosed by synovial macrophages, while PRG4 inhibits the phagocytic function of macrophages. Phagocytosis of CII-MSU crystals results in release of IL-1 and IL-8, and upregulates TLR2 and TLR4 expressions on macrophages, which leads to a positive feedforward effect, as DAMPs in the joint promote further inflammation. PRG4, on the other hand, inhibits IL-1 and IL-8 release MSU-stimulated macrophages and binds TLR2 and TLR4 receptors, thereby attenuating their activation by DAMPs.

**Table 1.** In vivo efficacy of native and recombinant PRG4/lubricin in pre-clinical models of post-traumatic osteoarthritis (PTOA) and the pharmacokinetic profile of recombinant human PRG4 as a potential biologic therapeutic for PTOA.

Study	Model and Treatment(s)	Outcome(s)
Flannery et al. [43]	Rat meniscectomy; I.A. recombinant human lubricin construct with one third KEPAPTT-like sequence 3× week or 1× week for 4 weeks.	Both treatments reduce cartilage degeneration and total joint scores.
Jay et al. [44]	Rat ACLT; I.A. recombinant full-length lubricin, HSL or HSFL 2× week for 4 weeks.	HSL reduces cartilage degeneration scores; HSL and HSFL reduce uCTXII levels, and all lubricins enhance aggrecan synthesis.
Teeple et al. [45]	Rat ACLT; I.A. hyaluronan, HSFL or hyaluronan + HSFL 2× week for 4 weeks.	HSFL alone or hyaluronan + HSFL reduce radiographic and cartilage degeneration scores with no effect by hyaluronan alone.
Jay et al. [46]	Rat ACLT; I.A. HSL once on day 7 post-surgery and analysis at 10 weeks.	HSL enhances aggrecan synthesis, reduces uCTXII levels, and improves weight bearing in injured joints.
Elsaid et al. [47]	Rat ACLT + forced exercise; HSFL on day 7 post-surgery and analysis at 5 weeks	Forced exercise aggravates cartilage damage and increases uCTXII excretion; HSFL treatment protects against ACLT + forced exercise cartilage damage.
Elsaid et al. [48]	Rat ACLT; I.A. IL-1ra 4× week for one week; I.A. IL-1ra + rhPRG4 once on day 7 post-surgery and analysis at 5 weeks.	IL-1ra reduces synovial inflammation and increases lubricin levels in SF; rhPRG4 and IL-1ra synergistically reduce chondrocyte apoptosis.
Waller et al. [49]	Minipig DMM; I.A. rhPRG4, hyaluronan or rhPRG4 + hyaluronan 3× week for one week and analysis at 26 weeks post-surgery.	rhPRG4 reduces medial tibial plateau macroscopic cartilage damage, uCTXII levels, SF, and serum IL-1β
Hurtig et al. [50]	Minipig ACLT; I.A. ^131^ I-rhPRG4 once with analysis at 10 min, 24, 72 h, 6, 13 and 20 days.	rhPRG4 joint elimination kinetics follows a two-compartment model with t_1/2β_ of 4.81 days.

ACLT: anterior cruciate ligament transection; DMM: destabilization of the medial meniscus; HSL: human synoviocyte lubricin; HSFL: human synovial fluid lubricin; I.A.: intra-articular; IL-1β; interleukin-1 beta; IL-1ra: interleukin-1 receptor antagonist; rhPRG4: recombinant human proteoglycan 4; SF: synovial fluid; uCTXII: urinary C-terminal crosslinked telopeptide type II collagen.

**Table 2.** Summary of studies on the PRG4/CD44/PP2A circuit in gout pathogenesis. Key findings supporting a biological role of CD44 as a urate crystal phagocytic receptor, with its ligand PRG4 as an anti-inflammatory biologic in acute gout, and a mechanism of action mediated by enhancing PP2A activity.

Study	Models and Treatment(s)	Outcome(s)
Qadri et al. [86]	TLR2 ligand stimulated human THP-1 macrophages; effect of hyaluronan or CD44-directed antibody. TLR2 ligand stimulated murine Cd44+/+ and Cd44−/− BMDMs.	TLR2 ligand enhances CD44 density on macrophages with no effect on TLR density. Hyaluronan and CD44 antibody reduce IL-1b and TNF via inhibition of the NF-kB pathway. PP2A mediates hyaluronan’s and anti-CD44 antibody anti-inflammatory effects. Lower TNF secretion with Cd44−/− BMDMs.
Bousoik et al. [81]	MSU crystal phagocytosis and IL-1b release by Cd44+/+ and Cd44−/− BMDMs. CD44 antibody treatment in MSU crystal stimulated human THP-1 macrophages. Peritoneal model of acute gout in Cd44+/+ and Cd44−/− mice; Cd44+/+ mice ± I.P. anti-CD44 antibody.	MSU crystal phagocytosis and IL-1b release are lower in Cd44−/− BMDMs. The antibody induces CD44 ECD shedding, reduces MSU crystal and ECD internalization, enhances PP2A activity, and suppresses NLRP3 inflammasome activation. Neutrophil and Ly6Chi monocyte recruitment and IL-1b secretion are suppressed in Cd44−/− mice; I.P. anti-CD44 antibody reduces markers of inflammation.
Qadri et al. [82]	MSU crystal phagocytosis by THP-1 macrophages ± rhPRG4. MSU crystal phagocytosis and IL-1b release by Prg4+/+ and Prg4−/− peritoneal macrophages. Rat knee model of acute gout ± I.A. rhPRG4.	rhPRG4 reduces MSU crystal phagocytosis, NLRP3 inflammasome activation and IL-1b release in a CD44-dependent manner. Prg4−/− peritoneal macrophages show greater MSU crystal uptake and IL-1b release. I.A. rhPRG4 reduces SF lavage MPO activity and normalizes weight bearing.
Elsaid et al. [83]	XOR expression and urate secretion by IL-1b + GM-CSF stimulated murine BMDMs ± rhPRG4. XOR expression in synovial macrophages in Prg4-deficient mice ± I.A. IL-1b	rhPRG4 reduces XOR expression and urate secretion. XOR expression in Prg4-deficient macrophages is higher and confined to CD86+ pro-inflammatory macrophages. CD206+ anti-inflammatory macrophages have minimal XOR expression.
Elsayed et al. [84]	MSU crystal phagocytosis by THP-1 monocytes and downstream signaling ± rhPRG4 or IL-1ra. MSU crystal phagocytosis by gout and normal PBMCs ± rhPRG4 or IL-1ra. Peritoneal model of acute gout in Prg4-deficient and Prg4-competent mice; efficacy of I.P. rhPRG4 in Prg4-deficient mice.	rhPRG4, but not IL-1ra, reduces MSU crystal phagocytosis. Both treatments reduce IL-1b release. rhPRG4 activates PP2A in monocytes. MSU crystal phagocytosis is higher in monocytes from gout patients compared to normal subjects, and both rhPRG4 and IL-1ra are efficacious in reducing IL-1b release. Prg4-deficient mice demonstrate ineffective resolution of inflammation (higher neutrophils and IL-1b levels), and rhPRG4 treatment promotes the effective resolution of inflammation.
Qadri et al. [85]	PP2A activity following MSU crystal phagocytosis by THP-1 monocytes; impact of PP2A catalytic subunit silencing on IL-1b release in MSU-challenged monocytes. Impact of the PP2A-activating drug, fingolimod phosphate, on NLRP3 inflammasome activation and IL-1b release by monocytes.	PP2A activity is reduced following MSU crystal phagocytosis. PP2A silencing increases IL-1b release in MSU-challenged monocytes. Fingolimod phosphate activates PP2A in MSU-challenged monocytes, and inhibits NLRP3 inflammasome activation and IL-1b release.

BMDMs: bone marrow-derived macrophages; ECD: extracellular domain; GM-CSF: granulocyte macrophage colony-stimulating factor; I.A.: intra-articular; I.P.: intra-peritoneal; IL-1β: interleukin-1 beta; IL-1ra: inteleukin-1 receptor antagonist; MPO: myeloperoxidase; MSU: monosodium urate; PBMCs: peripheral blood mononuclear cells; PP2A: protein phosphatase 2A; TLR2: toll-like receptor 2; TNF: tumor necrosis factor; XOR: xanthine oxidoreductase.
